# Unraveling the complex pathophysiology of white matter hemorrhage in intracerebral stroke: A single‐cell RNA sequencing approach

**DOI:** 10.1111/cns.14652

**Published:** 2024-03-03

**Authors:** Lisha Ye, Xiaoyan Tang, Jun Zhong, Wenfeng Li, Ting Xu, Chao Xiang, Jianjun Gu, Hua Feng, Qianqian Luo, Guohua Wang

**Affiliations:** ^1^ Department of Neurophysiology and Neuropharmacology, Institute of Special Environmental Medicine and Co‐innovation Center of Neuroregeneration Nantong University Nantong Jiangsu China; ^2^ Department of Neurosurgery, Key Laboratory of Neurotrauma, Southwest Hospital Third Military Medical University (Army Medical University) Chongqing China; ^3^ Department of Neurosurgery Zhengzhou University People's Hospital (Henan Provincial People's Hospital) Zhengzhou Henan China

**Keywords:** collagenase IV, heterogeneity, intracerebral hemorrhage, single‐cell RNA sequencing, white matter hemorrhage, white matter injury

## Abstract

**Aim:**

This study aims to elucidate the cellular dynamics and pathophysiology of white matter hemorrhage (WMH) in intracerebral hemorrhage (ICH).

**Methods:**

Using varying doses of collagenase IV, a consistent rat ICH model characterized by pronounced WMH was established. Verification was achieved through behavioral assays, hematoma volume, and histological evaluations. Single‐cell suspensions from the hemorrhaged region of the ipsilateral striatum on day three post‐ICH were profiled using single‐cell RNA sequencing (scRNA‐seq). Gene Ontology (GO) and gene set variation analysis (GSVA) further interpreted the differentially expressed genes (DEGs).

**Results:**

Following WMH induction, there was a notable increase in the percentage of myeloid cells and oligodendrocyte precursor cells (OPCs), alongside a reduction in the percentage of neurons, microglia, and oligodendrocytes (OLGs). Post‐ICH WMH showed homeostatic microglia transitioning into pro‐, anti‐inflammatory, and proliferative states, influencing lipid metabolic pathways. Myeloid cells amplified chemokine expression, linked with ferroptosis pathways. Macrophages exhibited M1 and M2 phenotypes, and post‐WMH, macrophages displayed a predominance of M2 phenotypes, characterized by their anti‐inflammatory properties. A surge in OPC proliferation aligned with enhanced ribosomal signaling, suggesting potential reparative responses post‐WMH.

**Conclusion:**

The study offers valuable insights into WMH's complex pathophysiology following ICH, highlighting the significance and utility of scRNA‐seq in understanding the cellular dynamics and contributing to future cerebrovascular research.

## Graphical abstract

The standard model for white matter hemorrhage (WMH) was established by intracerebral injection of collagen IV. The WMH model was evaluated by measuring hematoma volume, conducting behavioral tests, and performing histological staining. To gain a more comprehensive understanding of the cellular landscape of WMH, differentially expressed genes (DEGs) in certain types of brain cells were interpreted using single‐cell RNA sequencing (scRNA‐seq) and analyzed with Gene Ontology (GO) and gene set variation analysis (GSVA). This approach offers unprecedented granularity in interpreting the molecular mechanisms underlying WMH.

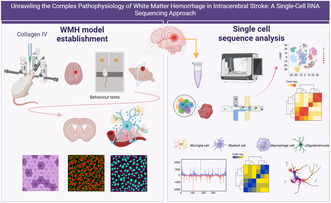

## 1 INTRODUCTION

Intracerebral hemorrhage (ICH) is a severe form of stroke with a growing incidence of high mortality and disability each year.^1^, ^2^ This medical condition is predominantly found in individuals over 40 years old with hypertension or vascular malformation.^3^ Within the umbrella of ICH, white matter hemorrhage (WMH) has become particularly concerning, as it's emerging at younger ages and accounts for significant cerebral apoplexy and other neurological complications.^4^, ^5^ White matter (WM) consists of a myelin sheath formed by mature oligodendrocytes around axons, playing a vital role in signal transmission that connects various parts of gray matter.^6^ WM is especially susceptible to ICH, notably within the capsule.^7^ Damage to the myelin sheath leads to structural lesions and transient inflammatory reactions,^8^ causing sensory and advanced cognitive dysfunction in subsequent injury stages. This emphasizes the profound damage ICH can inflict, particularly in the WM area.^7^ The pathophysiology of WMH is multifaceted, encompassing complex primary and secondary mechanisms, such as pronounced inflammatory responses.^9^, ^10^ The aftermath of hemorrhage includes activation of microglia cells, infiltration of inflammatory cells, and cytokine release, contributing to further WM injury (WMI).^11^, ^12^ These underlying processes present challenges for modeling and treatment, due to the condition's heterogeneity,^13^, ^14^ and a comprehensive understanding remains elusive.

The ongoing investigation into WMH encounters two principal obstacles. First, the lack of standardized models for cerebral WMH hampers progress.^10^ The search for a stable, repeatable animal model that accurately reflects human WMH is urgent, as it can aid in understanding and identifying therapeutic targets.^15^ To that end, our research is exploring an inflammation‐mediated WMH model using collagenase,^16^ facilitating precise and quantitative modeling. Second, there is a scarcity of evaluations focusing on specific cell types in hemorrhagic brain regions, particularly in rat models of cerebral hemorrhage.^17^ Ensuring model consistency, considering factors like age, sex, and genetic background of animals, requires concerted efforts.^18^ Recent advancements, such as single‐cell sequencing (SCS), offer fresh promise.^19^, ^20^ By enabling high‐throughput analysis at individual cell levels, SCS provides exceptional insights into cellular heterogeneity and disease mechanisms,^21^ thus enhancing our understanding of ICH and offering new avenues for therapy.

Despite the clinical and genetic heterogeneity of ICH, numerous studies have shown that gene expression changes at the site of hemorrhage are focused on common genes and pathways.^14^, ^22^ Meanwhile, ICH's impact on molecular networks, including both pro‐inflammatory and anti‐inflammatory systems, offers insights into secondary injury mechanisms.^21^, ^23^ However, direct assessments of specific cell types in WMI models are still lacking. In this study, we undertake a focused investigation into the cellular responses in WMH, using cutting‐edge techniques like single‐cell RNA sequencing (scRNA‐seq). By injecting collagenase IV into the right striatum of rat brains, we've established a standardized WMH model. Through scRNA‐seq, Gene Ontology (GO) analysis, and gene set variation analysis (GSVA), we've successfully mapped differentially expressed genes and visualized the data. Our findings reveal dynamic cell interactions post‐WMH, highlighting changes in microglial states and uncovering lipid metabolism pathways. This investigation lays a groundwork for further exploration, holding great promise for advancing our understanding and treatment of WMH.

## 2 MATERIALS AND METHODS

### 2.1 Animal experimentation

Male Sprague–Dawley rats for well‐documented brain anatomy and physiological responses that closely mimic human pathologies, aged 8 weeks and weighing between 220 and 230 g, were sourced from Nantong University's Experimental Animal Center, Nantong, China. They were housed under specific pathogen‐free conditions within stainless steel cages, maintaining a stable environment at 26°C and relative humidity ranging from 55% to 60%. A 12‐h light/dark cycle was adhered to, and the rats had ad libitum access to food and water. All protocols were strictly in accordance with the Animal Management Rules of the Ministry of Health of China, the experimental animal management standards of Nantong University, and were approved by the Animal Ethics Committees of Nantong University (No. S20200316‐021). The rats were randomly assigned into two primary groups, namely, sham operation and WMH (ICH‐str), with the latter subdivided into three categories based on varying doses of collagenase type IV. Each group contained no fewer than six rats.

### 2.2 Establishment of the WMH model

The development of the WMH was meticulously carried out in accordance with the ICH model.^16^ Rats were securely placed on a stereotactic apparatus (Model 68002, Steeling, USA) and anesthetized with a mixture of 1.5% isoflurane, 30% O_2_, and 68.5% N_2_O. The surgical process began with disinfection using iodine and alcohol, followed by a scalp incision to expose the skull. The injection procedures, site determination, and collagenase dosage were in accordance with established protocols.^16^ Specifically, a 1 mm burr hole was created over the right basal ganglia, located 3.5 mm lateral and 0.2 mm anterior to the bregma. Collagenase IV (C5138, Sigma, USA) was prepared in varying concentrations (0.25, 0.5, or 1 mU/Kg) and promptly dissolved in 2 μL of sterile saline. This solution was then drawn into a Hamilton microsyringe, which was affixed to the stereotactic apparatus (Stoelting Co. Ltd., USA). For the control group, 2 μL of sterile saline was used. The microsyringe, connected to an automated injector, delivered the solution at a rate of 0.4 μL/min. After injection, the needle tip remained embedded in the brain tissue (5.5 mm below the skull surface) for 5 min, and then for an additional 5 min at a depth of 3.5 mm. The microsyringe was subsequently retracted gently, and the burr hole was sealed using bone wax. Finally, it was determined whether the model was successfully constructed by checking limb movement.

### 2.3 Neurological evaluations

#### 2.3.1 Corner turning test

The corner turning test was conducted as described in previous research,^24^ and the procedure involved evaluating the rats' turning behavior in a specially designed corner apparatus. Briefly, the corner was constructed of two flat panels at a 30‐degree angle, with a small slit at the junction to attract rats. Repeated trials (10 times each rat) ensured accurate assessment. If a rat turned to the ventral side (standing without hind legs), the results were disregarded and reattempted during the final phase of the test.

#### 2.3.2 Modified Neurological Severity Score (mNSS)

The mNSS score incorporates assessments of motor function, sensory function, balance, reflexes, and abnormal movements. The scoring system attributes one point for each task or reflex the rat fails to complete, providing a comprehensive evaluation of sensorimotor impairment as previously described.^25^

### 2.4 Brain hemorrhage volume calculation

At 3 and 7 days after modeling, rats were perfused through the left ventricle with 0.9% saline, followed by 4% paraformaldehyde. The brains were then removed and post‐fixed for 24 h in 4% paraformaldehyde, followed by 20% and 30% sucrose gradient dehydration. Afterward, the brain tissues were sectioned into frozen coronal sections (30 μm thick) using a cryostat (Leica, Bensheim, Germany). The sections were mounted and Nissl stained for histological assessment of the injury. The adjacent free‐floating sections were processed for immunohistochemical analysis. ImageJ image processing and analysis software was used to slice the cerebral hemorrhage surface of rats. The formula used to calculate the percentage of cerebral ischemia volume is Hemorrhage volume ratio = (sum of red hemorrhage area of each section)/(sum of brain section area of each section) × 100%.

### 2.5 Nissl staining

The tissue sections underwent sequential processing, beginning with immersion in chloroform for 30 min and acetone for 15 min. They were then rehydrated in a graded series of ethanol concentrations (100%, 95%, and 70%) for 30 s each, followed by two brief rinses in distilled water. After staining with cresyl violet (Sigma‐Aldrich) for 20 min, the sections were rinsed and dehydrated in a reverse graded ethanol series, cleared in chloroform for 5 min, and made transparent with xylene. They were subsequently mounted using neutral balata. Imaging was performed with a Leica DM4000B microscope (Germany).

### 2.6 Hematoxylin–eosin (HE) staining

The brain tissue specimens were fixed in a 4% paraformaldehyde solution. Following fixation, the tissues underwent a graded dehydration process using an ethanol series and were subsequently cleared in xylene. The specimens were sectioned at a thickness of 30 μm and adhered to anti‐adhesive slides. HE staining was employed using the kit from SERVICEBIO (G1005, China). Post‐staining, the sections were dehydrated through a gradient of ethanol from low to high concentrations, followed by clearing in xylene. The slices were then sealed with neutral balsam and examined under a Leica DM4000B microscope.

### 2.7 Luxol fast blue (LFB) staining

Coronal tissue sections were subjected to Luxol Fast Blue (LFB) staining, as prescribed by the manufacturer (American Mastertech, USA), to evaluate myelin damage.^26^ The slides were immersed in a pre‐warmed LFB solution at 60°C for an hour. After this, the sections were rinsed with water and differentiated using a lithium carbonate solution for 30 s, then treated with 70% ethanol until the demarcation between gray and white matter was discernible and the nuclei were decolorized. Following an ethanol dehydration step, the sections were mounted using neutral balata and prepared for microscopic observation.

### 2.8 Immunofluorescent staining

Coronal tissue sections were incubated with one of the primary antibodies: anti‐MBP antibody (1:300, ab40390, Abcam, UK), SMI‐32 (1:200, NE1023, Millipore, USA), Neuron (1:100, Millipore, MAB377, USA), or Iba 1 (1:1000, Wako, 019–19,741, USA) in a blocking solution at 4°C overnight. Subsequently, sections were exposed to secondary antibodies, specifically DAM‐Alexa488 and DAR‐Alexa555, and incubated at room temperature for 2 h. Fluorescent images were captured using a confocal laser scanning microscope (TCS SP8, Leica, Germany). The quantitative analysis involved assessing the immunostaining intensity of both MBP and SMI32 antibodies, as well as enumerating target immunopositive cells using ImageJ software.^26^ A total of three randomly chosen microscopic fields from each of the three sequential sections were examined per mouse brain. White matter damage was quantitatively determined by calculating the average intensity ratio of SMI32 to MBP.

### 2.9 Preparation of single‐cell suspensions from rat brain tissue for library generation and sequencing

**FIGURE 1:**
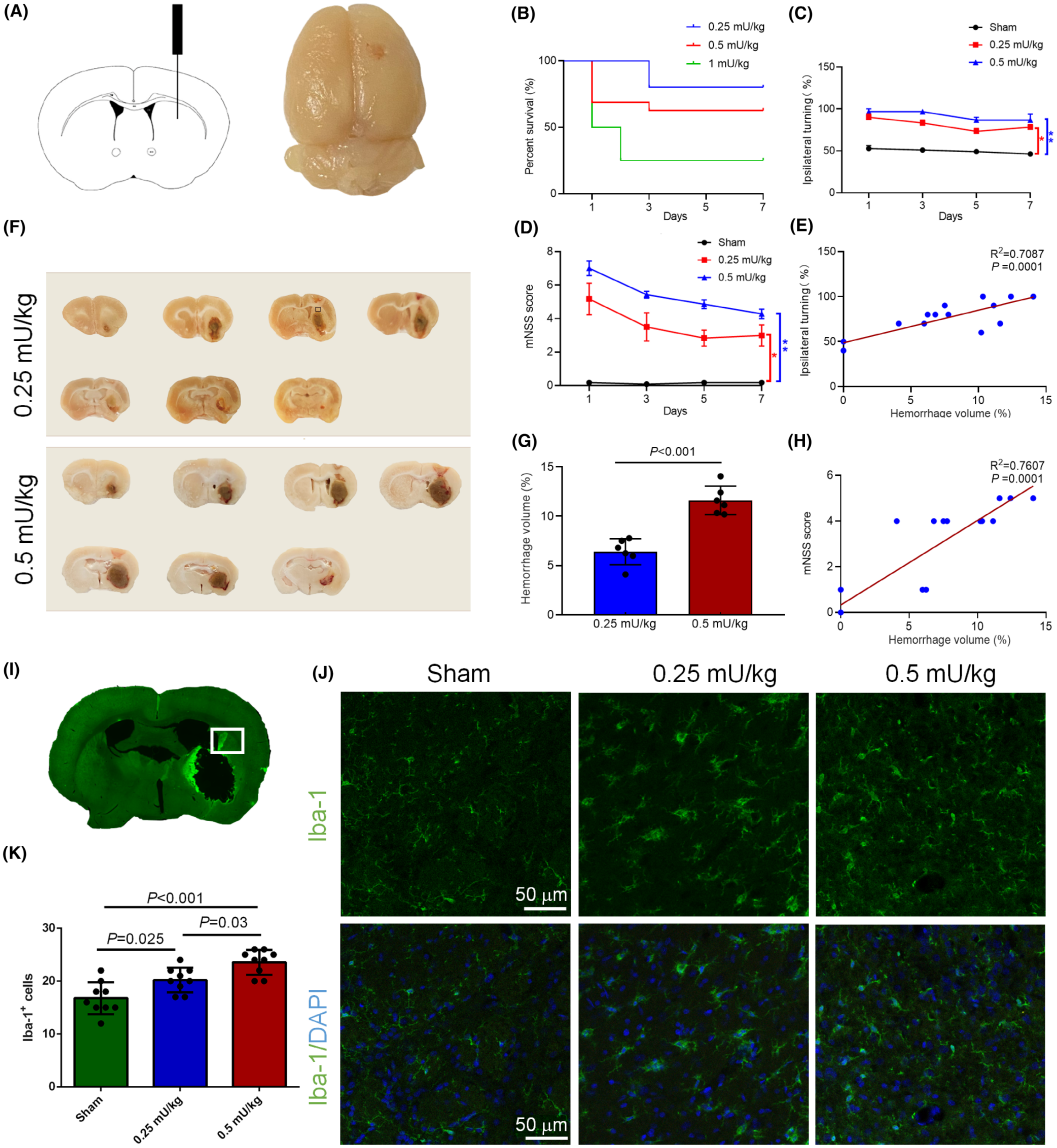
Quantitative analysis of neurological function and hemorrhagic volumetrics in a collagenase IV‐induced WMH rat model. (A) Schematic representation of precise stereotaxic administration of collagenase IV into the striatal region, facilitated by a stereotaxic apparatus for rat cranial immobilization. (B) Mortality rates observed at varying dosages of collagenase IV. (C) Quantitative representation of ipsilateral turning in experimental rats at days 1, 3, 5, and 7 post‐WMH induction. (D) Modified Neurological Severity Scores (mNSS) of rats on days 1, 3, 5, and 7 following WMH occurrence. (E) Correlational analysis delineating the relationship between the volumetric extent of the hemorrhage and ipsilateral turning behavior. (F) Representative images illustrating the extent of striatal hemorrhage across various coronal brain sections at different collagenase dosages. The regions of brain tissue selected for scRNA‐seq are represented by the black box. (G) Statistical evaluations of hemorrhagic volume alterations after WMH induction at varying collagenase concentrations. (H) Correlational assessment linking hemorrhagic volume to the corresponding mNSS values. (I) Representative full‐brain coronal sections depicting Iba‐1 immunofluorescence staining post‐WMH. (J) Immunofluorescent imaging reveals increased prevalence and morphological alteration of microglia/macrophage cells, evidenced by Iba‐1 staining, subsequent to WMH. Morphological changes, including enlarged cell bodies and shortened extensions, were particularly pronounced in the high‐dose (0.5 mU/kg) WMH group. (K) Quantitative analysis of microglia/macrophage prevalence in the perihemorrhagic striatal regions at varying collagenase dosages. Data are presented as mean ± SEM; *n* = 6–11 per experimental group. *p < 0.05, **p < 0.01 relative to the indicated comparison group. The scale bar represents 50 μm.

Sample collection for single‐cell RNA sequencing (scRNA‐seq) was performed 3 days post‐modeling, with rats undergoing isoflurane inhalation. Three brain tissues of each group were cut into small pieces measuring 0.3 cm × 0.3 cm (see Figure 1F) on ice. A detailed procedure of collection, handling, and preparation of brain tissues was followed, including precise cutting and shipment to Shanghai Genechem Co., LTD for scRNA‐seq analysis. Briefly, the tissue underwent dual enzymatic digestion: initially with 0.125% trypsin solution (Life Technologies Inc., Grand Island, NY) followed by subsequent digestion using a 0.1% collagenase solution (Sigma, St. Louis, MO). After pooling the digestion mixtures, the resultant solution was centrifuged to harvest the brain cells. The resulting single‐cell suspension was then sieved through a 40 μm nylon mesh to discard any cellular aggregates. The isolated cells were subsequently rinsed and resuspended in phosphate‐buffered saline devoid of calcium and magnesium and supplemented with 0.1% bovine serum albumin. The cell viability was ascertained using Trypan blue staining (Bio‐Rad, Irvine, CA). For the library generation intended for the 10× Genomics system, only cells exhibiting a viability of 90% or greater were selected. Adhering to the manufacturer's protocol, these cells were processed for barcoded cDNA library synthesis and the generation of genetically engineered models (GEMs). As a final quality assurance step, all prepared libraries were evaluated using a Fragment Analyzer 2100 (Agilent Technologies, Carpinteria, CA) and were subsequently sequenced on the Illumina NovaSeq 6000 platform (Illumina, San Diego, CA).

### 2.10 Quality control, cluster identification, and subcluster analysis of scRNA‐seq data

The raw sequencing data were processed utilizing the Cell Ranger software pipeline (version 3.1.0) provided by 10× Genomics. Raw binary base call files were demultiplexed employing the “cellranger mkfastq” function. The “cellranger counts” feature, under default parameters, managed individual read alignments and generated transcript counts for each specimen. The unique molecular identifier (UMI) count matrix was subsequently loaded into the Seurat R package (version 3.2.0) for further analysis. Integral quality control was executed for each generated library. Specifically, cells were discarded if they exhibited fewer than 500 genes, had a mitochondrial gene content surpassing 10%, or if their UMI/gene metrics deviated beyond a two‐fold standard deviation from the mean, presupposing a Gaussian distribution for UMI/gene counts per cell. To address batch effects inherent to the scRNA‐seq data, the “batchelor” R package was utilized, targeting mutual nearest neighbors (MNN) for analysis. After quality assurance, the Seurat R package facilitated the standardized assessment of the scRNA‐seq dataset. Library size normalization was applied to the filtered matrix, securing the normalized counts. Subsequently, high‐variance genes within individual cells were identified. Initially, we performed dimensionality reduction on the high‐variance genes identified in individual cells. This was accomplished using principal component analysis (PCA), which reduced the complexity of the data while preserving the most significant variations. After PCA, we employed a graph‐based clustering approach using the “Find Clusters” function in the Seurat package. This method constructs a shared nearest neighbor (SNN) graph based on the PCA results. Cells are then grouped based on their connectivity in this graph, effectively clustering cells with similar gene expression profiles. The clusters were visualized using t‐distributed Stochastic Neighbor Embedding (tSNE), a technique that allows the high‐dimensional data to be represented in two dimensions, facilitating the identification of distinct cell groups. The “FindAllMarkers” function in Seurat delineated marker genes for each cluster, contrasting a cluster's positive markers against the entirety of the cell population. Gene Ontology (GO) analysis rooted in the hypergeometric distribution was executed in R. Following this primary annotation, cell subsets exhibiting adequate cell counts were extracted employing the “SubsetData” strategy. These chosen cells underwent re‐clustering via tSNE and were subsequently annotated using dominant cell markers, post utilization of the “Find Clusters” and “Find All Markers” functions.

### 2.11 Functional pathway enrichment analysis

Gene set enrichment analyses were conducted using GSVA, drawing upon C2, C5, and GO Molecular Signatures Database (MSigDB) gene sets, to discern activated functional pathways within the cell clusters. Pathways with adjusted *p*‐values exceeding 0.05 were excluded from subsequent investigations. Visual representations of the up‐ and down‐regulated pathways were furnished through bar plots or heatmaps.

The GSVA and MSigDB gene sets were used to identify key pathways involved in WMH pathophysiology. These analyses helped us understand the molecular mechanisms underlying the observed cellular changes, particularly in relation to inflammation and neuronal damage.

### 2.12 Single‐cell trajectory analysis

Monocle software facilitated the exploration of single‐cell trajectory data to delineate developmental shifts. Employing Seurat's highly variable genes, cells were organized in a pseudotemporal sequence. The initial cell state within this pseudotime was ascertained via the “orderCells” methodology and designated as the “root state argument”. Dimensionality reduction was achieved through “DDRTree”. Differentially expressed genes across pseudotime were determined using the “differentialGeneTest”, and the results were exhibited through “plot cell trajectory”.

### 2.13 Analytical procedures and statistical analysis

Marker genes in the scRNA‐seq datasets were pinpointed using the “FindAllMarkers” function from the Seurat package, setting a minimum log‐fold change threshold at 0.25. *p*‐values were computed via the Wilcoxon rank‐sum test. For evaluating biological processes and canonical pathways across distinct clusters, GSVA was employed in tandem with the MSigDB. In addition, the top ten prominently expressed marker genes were incorporated into a biological process enrichment assessment using DAVID. Visualization of the results was facilitated through the R package, leveraging tools such as ggplot2 and pheatmap.

Data analysis was systematically executed utilizing the Prism 8.0 software suite provided by GraphPad. Quantitative results were presented as mean ± standard error (SE). Statistical discrepancies between group means were evaluated utilizing one‐way analysis of variance (ANOVA) and multiple comparisons of Tukey's post hoc tests. For the analysis of behavioral test data, a two‐way repeated‐measures ANOVA was administered, succeeded by the application of Tukey's post hoc tests. All data is subject to tests for normality.

## 3 RESULTS

### 3.1 Impact of collagenase IV dosages on neurological function and volumetric percentage of WMH

Utilizing a microinjection needle, 2 μL of varying dosages of collagenase IV was methodically administered into the striatum (Figure 1A). The differential dosages of collagenase IV resulted in distinct mortality rates. Specifically, on the first day post‐surgery, only 50% of rats survived following the induction of WMH with a 1 mU/kg dose of collagenase. In the group administered a 0.5 mU/ kg dose, the survival rate was 68.75%. From the third to the seventh day, the dose groups receiving 0.25, 0.5, and 1 mU/kg had survival rates of 80%, 62.5%, and 25%, respectively (Figure 1B). Upon successful induction of the white matter hemorrhage model, neurosensorimotor alterations in the rats were closely observed. Subsequent assessments of the rats' neurological functionality were carried out through corner turning evaluations (Figure 1C) and mNSS scoring (Figure 1D) on post‐operative days 1, 3, 5, and 7. A pronounced neurological dysfunction was discerned in the rats on these days when juxtaposed with the sham group (*p* < 0.05). This observation elucidated the dose‐dependent ramifications of collagenase IV on both mortality and neurological functionality. In the aftermath of white matter hemorrhage induction via collagenase IV, a discernible variance in hematoma sizes was evident across brain tissues subjected to distinct collagenase doses in different coronal sections (Figure 1F). This hemorrhage volume showcased a dose‐dependent trend, with augmented collagenase dosages correlating to increased hemorrhage volumes when compared to the 0.25 mU/kg cohort (Figure 1G, *p* < 0.001). Notably, a positive correlation was observed between hemorrhage volume and both the corner turning test (Figure 1E) and mNSS scores (Figure 1H, all *p* < 0.001). Further, immunofluorescence staining of brain sections was executed using the Iba‐1 antibody to demarcate microglial cells, a specialized neuronal cell category in the cerebral system. In response to white matter hemorrhage and other inflammatory stimuli, these microglial cells underwent morphological alterations, exhibiting enlarged cell bodies and truncated processes, with some assuming amoeboid configurations (Figure 1I,J). A marked surge in microglial cell counts was observed in the collagenase‐treated groups in comparison to the sham group (Figure 1K, *p* < 0.05).

### 3.2 Neuronal and inflammatory cell expression and WMI in the striatum following WMH induced by varying doses of collagenase IV

**FIGURE 2:**
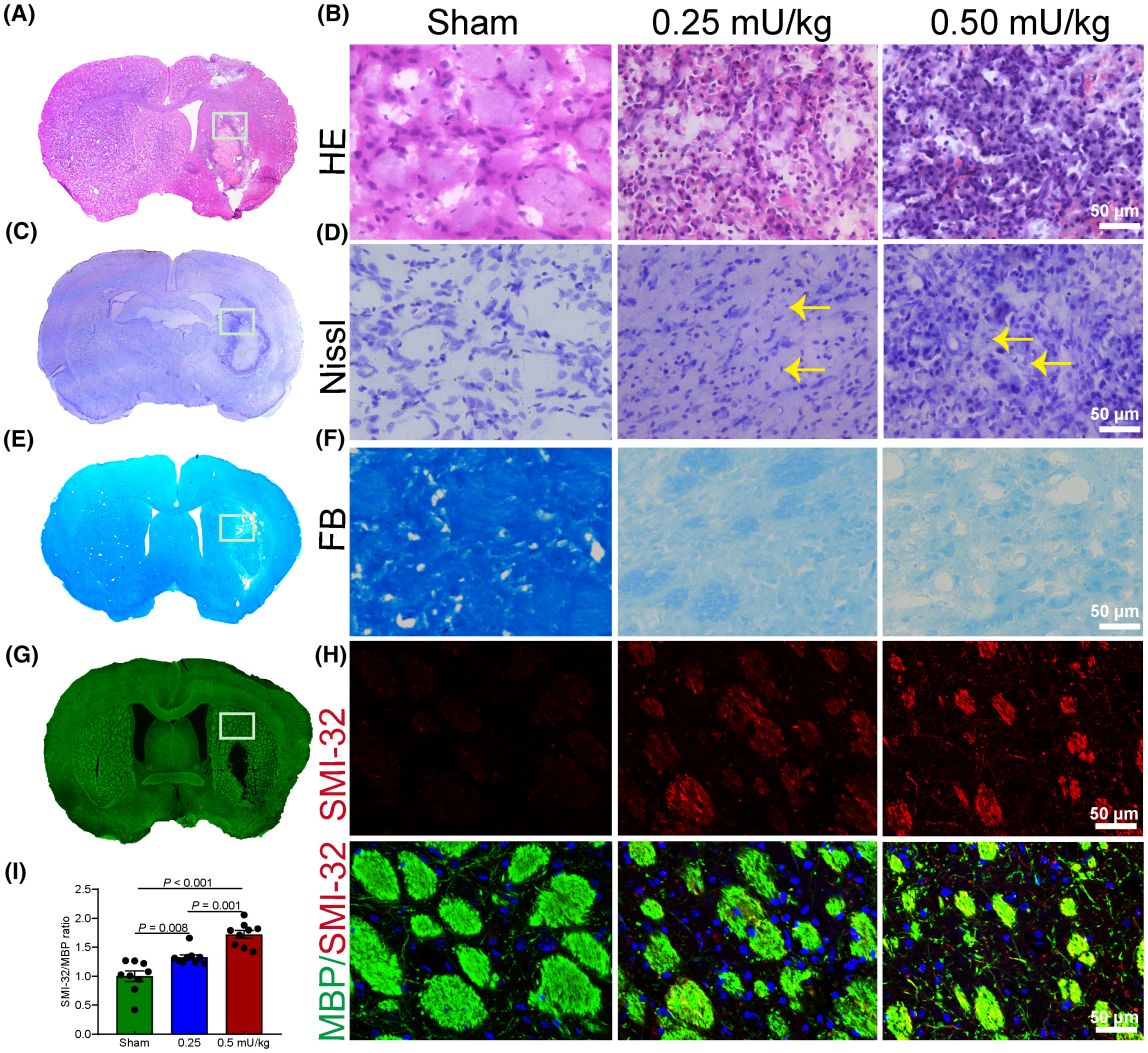
Differential activation of inflammatory cells and extent of white matter damage in response to varied collagenase IV dosages in striatal WMH model. (A) Representative site within brain tissue sections selected for HE staining. (B) Application of HE staining to delineate variations in inflammatory cell infiltration within striatal hemorrhagic zones across different collagenase IV dosage conditions. (C) Specified site for Nissl staining in the coronal sections of brain tissue. (D) Nissl staining was employed to visualize alterations in the inflammatory cell milieu surrounding striatal hemorrhagic sites induced by varying collagenase IV concentrations. Typical neuronal lysis and necrosis are indicated by yellow arrows. (E) Defined region within brain tissue sections selected for LFB staining. (F) Utilization of LFB staining to assess the extent of WMI in peri‐striatal areas following collagenase IV‐induced WMH in a dose‐dependent manner. (G) Designated site for the application of MBP immunofluorescence within brain tissue sections. (H) Double immunostaining of MBP and SMI‐32 unveils pathological transformations within the corpus callosum post‐collagenase IV‐induced striatal hemorrhage at different dosages. (I) Statistical quantification of alterations in SMI‐32/MBP fluorescence intensity ratios within the corpus callosum, consequent to striatal hemorrhage induced by varied collagenase IV dosages. Data are presented as mean ± SEM. The scale bar represents 50 μm.

Histological analysis revealed significant cellular changes post‐administration of collagenase IV. HE staining demonstrated an extensive infiltration of erythrocytes and inflammatory cells within the collagenase‐treated group (Figure 2A,B). Subsequent Nissl staining elucidated that collagenase‐induced WMH instigated inflammatory cell infiltration, accompanied by neuronal dissolution and necrosis within the striatum (Figure 2C,D). LFB staining was employed on brain tissue sections to delineate the striatal region for imaging. Marked demyelination was evident in the striatal region post‐white matter hemorrhage, leading to a characteristic blue‐light staining within the white matter area (Figure 2E,F). To further discern the repercussions of different collagenase dosages on the extent of WMI, dual immunofluorescence staining utilizing MBP and SMI‐32 was performed (Figure 2G–I). The SMI‐32 to MBP fluorescence intensity ratio served as an indicator of the degree of white matter repair. On the seventh day following white matter hemorrhage, selected brain sections were co‐labeled with MBP and SMI‐32. Subsequent quantification of the fluorescence intensity ratio of SMI‐32 to MBP within the corpus callosum's surrounding region revealed a significant elevation (Figure 2H,I, *p* < 0.01). This suggests that white matter hemorrhage augmented this ratio, underscoring a notable WMI.

### 3.3 Single‐cell profiling of striatal tissue following WMH induction

**FIGURE 3:**
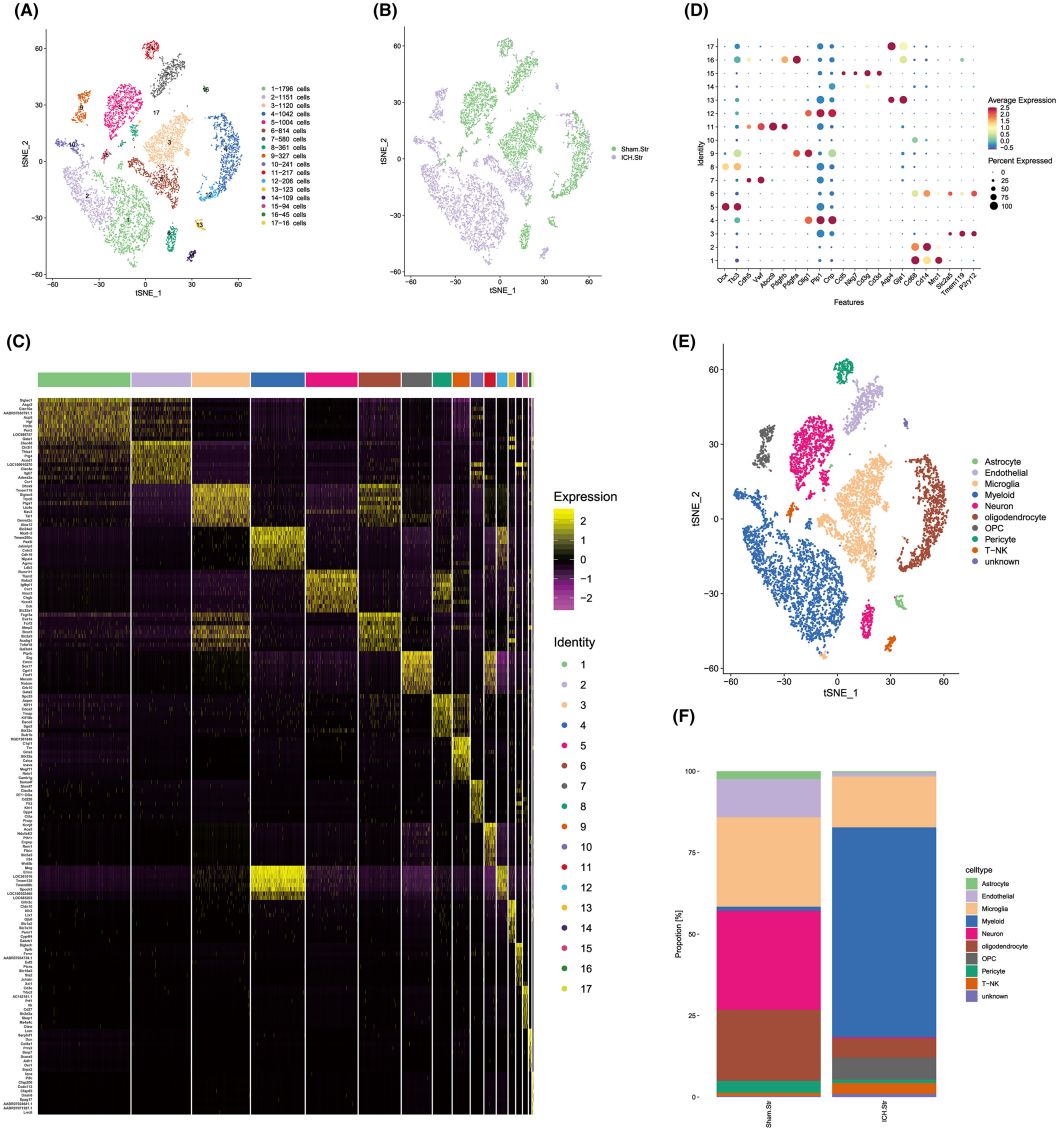
Comprehensive single‐cell transcriptomic profiling elucidates cellular heterogeneity in WMI following striatal hemorrhage. (A) Following fast MNN batch normalization, t‐distributed Stochastic Neighbor Embedding (t‐SNE) analysis was employed to visualize a composite of 4440 and 4806 cells isolated from the sham and WMH groups, respectively. Each cell is color‐coded to denote its assignment to a specific cluster. (B) Subsequent classification delineated 17 discrete cellular subclusters. (C) Identification and enumeration of the top 10 marker genes characterizing each subcluster. (D) Cellular categorization according to distinct marker gene expression profiles and Single R annotations. (E) Utilization of t‐SNE for high‐dimensional data reduction and cellular clustering. (F) Quantitative histogram representing the proportional distribution of various cellular phenotypes in the striatal region, both prior to and following the induction of WMH. Data are presented in a comprehensive format.

To elucidate cellular alterations post‐brain hemorrhage, striatal tissue samples were subjected to scRNA‐seq analysis. Striatal tissue, obtained from a consistent brain region, was prepared as single‐cell suspensions from both the WMH‐induced and sham groups. Following quality control procedures, which eliminated low‐quality and duplicated cells, 4440 cells from the sham group and 4806 cells from the WMH group were retained for analysis (Figure S1). Utilizing the fastMNN algorithm, the batch correction was executed, and subsequent tSNE clustering delineated all cells into 17 distinct subclusters (Figure 3A). This segregation was consistent across both sham and WMH samples (Figure 3B). The top 10 marker genes defining each subcluster were subsequently identified (Figure 3C). Based on these marker genes and SingleR annotations, cell identities were ascertained, leading to classifications such as neurons (*Ttc3* and *Dcx*), astrocytes (*Gja1* and *Aqp4*), oligodendrocytes (*Cnp* and *Plp1*), microglia (*P2ry12*, *Tmem119*, and *Slc2a5*), endothelial cells (*Vmf* and *Cdh5*), myeloid cells (*MRC1*, *CD14*, and *CD68*), T‐NK cells (*CD3E*, *CD3G*, and *NKG7*), and mural cells (*Pdgfrb* and *Abcc9*) (Figure 3D, Figure S1). Notably, the WMH group exhibited a significant augmentation in the percentage of myeloid cells, while displaying decreased counts of microglia, oligodendrocytes, and neurons (Figure 3E,F). This shift suggests a pronounced infiltration of peripheral myeloid cells in the WMH group. Concurrently, a surge in OPCs was evident. The WMH group further displayed a drastic reduction in neuronal proportion. Collectively, these observations underscore the extensive neuronal, microglial, and oligodendrocyte death at the hemorrhage site post‐WMH, accompanied by pronounced demyelination as a hallmark of severe brain injury.

### 3.4 Microglial heterogeneity analysis

**FIGURE 4:**
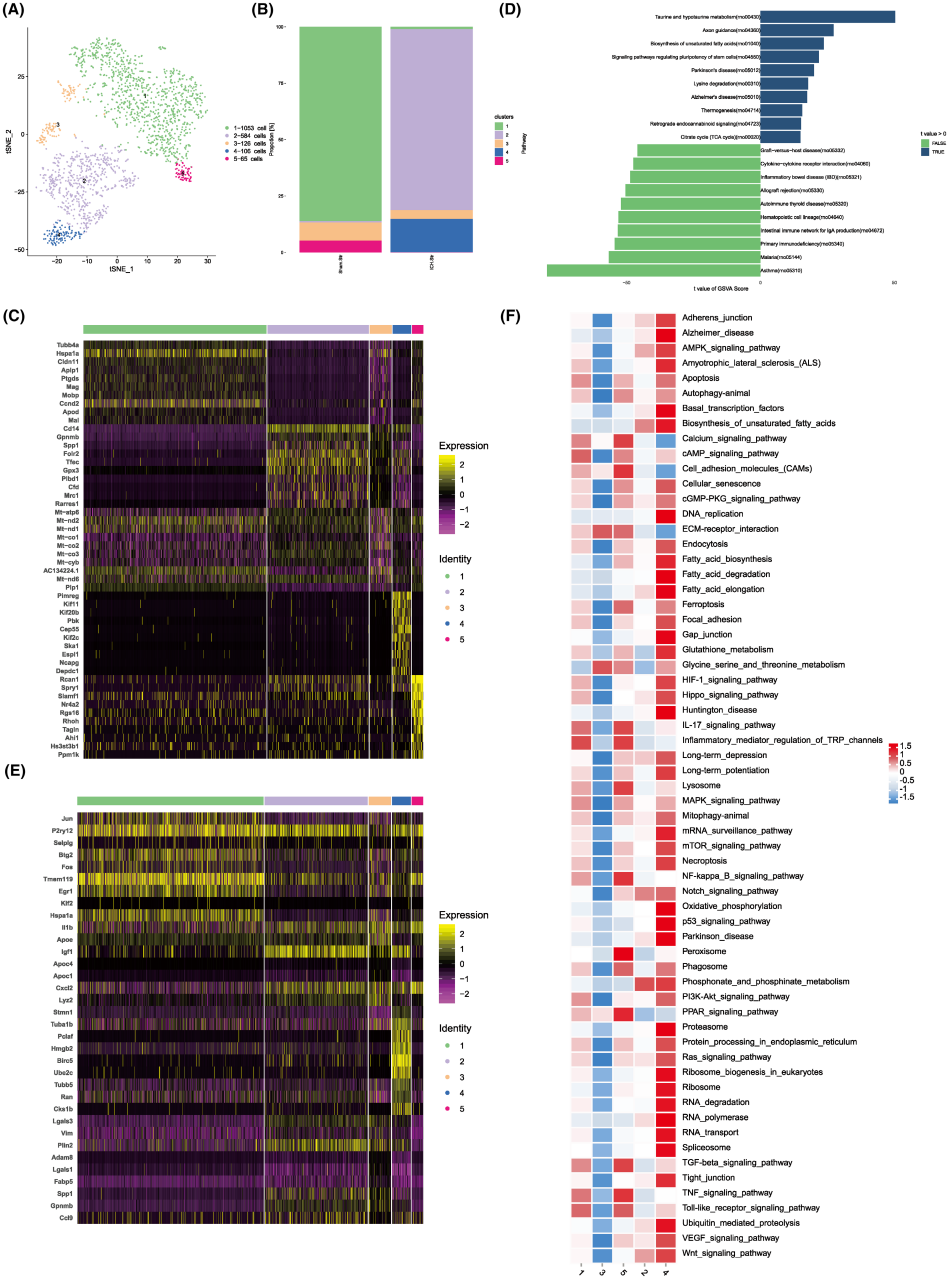
Comprehensive heterogeneity analysis of microglial subpopulations. (A, B) Discriminative clustering of microglial subtypes and respective proportions across various samples. (C) Enumeration and identification of the top 10 marker genes characterizing each of the five microglial subclusters. (D) Gene Set Variation Analysis (GSVA) was employed to elucidate differential pathway enrichment across samples. (E) Unambiguous identification of microglial cell subpopulations and their corresponding cellular phenotypes. (F) Heatmap visualization representing variances in biological processes across microglial clusters as determined by GSVA enrichment scores. Differential pathway enrichments among subclusters were scrutinized via GSVA.

Microglia, being the primary immune effector cells in the brain, swiftly react to brain injuries, thereby influencing the functionality of surrounding cells. To delve into the heterogeneity and diverse functionalities of microglia in response to WMI, we embarked on a sub‐cluster analysis. Our investigation segregated the microglia into five distinct clusters. Interestingly, clusters 1 and 5 predominantly stemmed from the sham group, while clusters 2 and 4 were chiefly evident in the WMH group (Figure 4A,B). This pattern suggests that clusters 2 and 4 represent microglia populations emerging post‐WMH. Subsequent exploration of the top 10 marker genes across each cluster (Figure 4C) showcased marked gene expression variations between the clusters from the two groups. GSVA discerned significant functional disparities between clusters, particularly in pathways such as taurine and hypotaurine metabolism, axon guidance, unsaturated fatty acid biosynthesis, asthma, malaria, and primary immunodeficiency (Figure 4D). Drawing on established literature,^22^ we deduced the phenotypes of the microglial sub‐clusters: clusters 1 and 5 were characterized by the heightened expression of homeostatic microglia markers like *P2ry12*; cluster 2 exhibited elevated levels of inflammatory markers such as *Igf1* and *Il1b*, while cluster 4 was enriched in dividing microglia markers, exemplified by *Birc5* (Figure 4E). In an overarching GSVA evaluation, it was discerned that, in contrast to the sham group, the WMH group manifested heightened activity in lipid metabolism pathways, encompassing fatty acid elongation, synthesis, degradation, and RNA degradation. Conversely, inflammatory signaling pathways, inclusive of IL‐17, TNF‐α, NF–κB, and Toll‐like receptors, were found to be inhibited (Figure 4E,F). Furthermore, under quantitative Real‐Time Polymerase Chain Reaction (qRT–PCR, Appendix S1), three and 7 days after WMH, M1 pro‐inflammatory microglia (highly expressing CD16, iNOS) are persistently expressed, while M2 anti‐inflammatory microglia reach their peak expression on day three post‐hemorrhage and then decline (Figure S2).

### 3.5 Myeloid cell heterogeneity analysis

**FIGURE 5:**
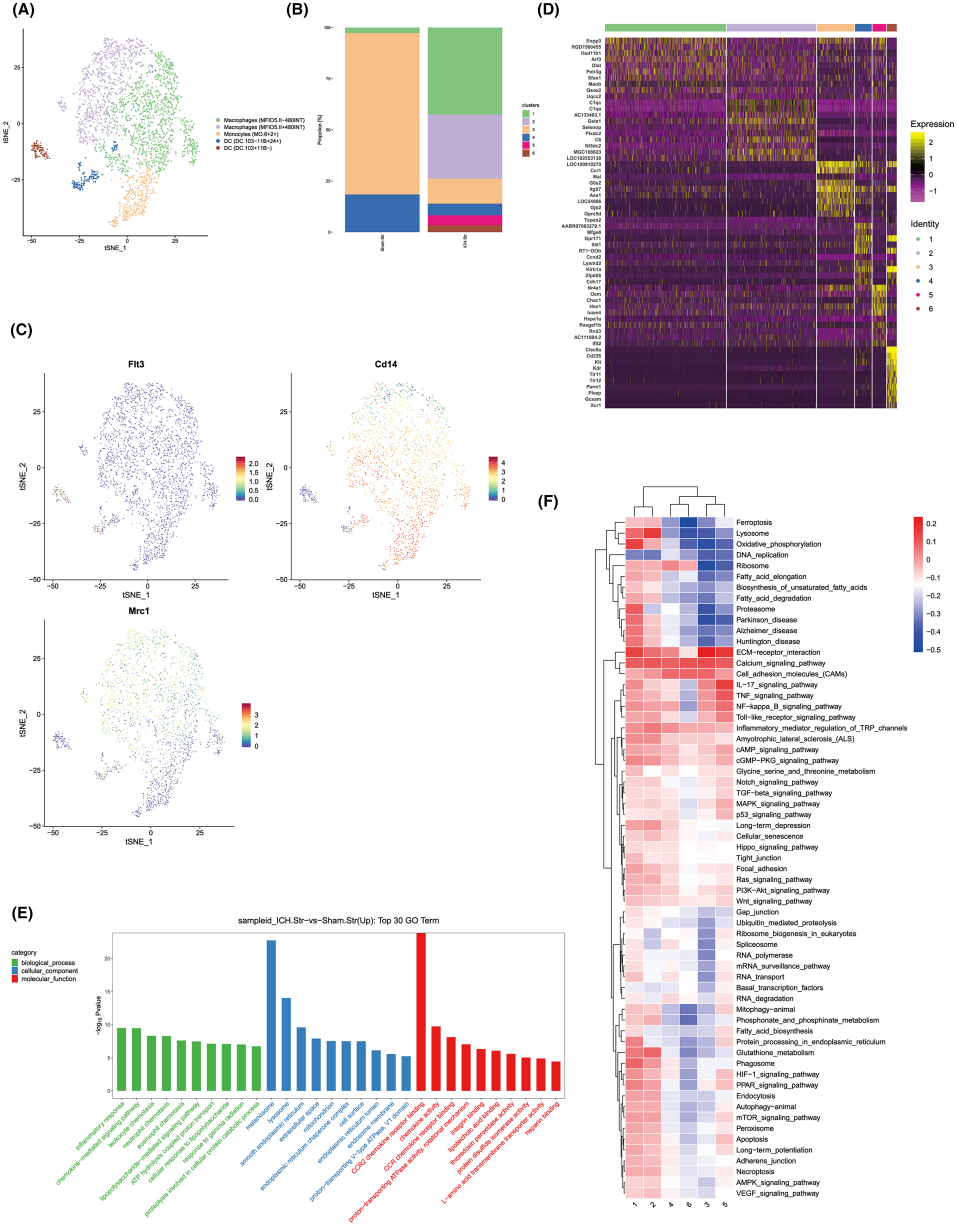
Detailed heterogeneity analysis of myeloid cell subpopulations. (A) Discriminative clustering and proportional distribution of myeloid cell subclusters across disparate samples. (B) Histogram representing the relative proportions of myeloid cell subclusters within the dataset. (C) Visualization of key marker genes pertinent to specific myeloid subtypes: FLT3 for dendritic cells (DC), MRC1 for macrophages, and CD14 for monocyte/macrophage lineage. (D) Enumeration and identification of the top 10 marker genes uniquely characterizing each of the six myeloid subclusters. (E) GO analysis illustrating differentially upregulated genes between WMH and sham conditions, focusing specifically on variations in myeloid cell expression. (F) GSVA is utilized to discern the differential gene expression across myeloid cell subsets.

From our single‐cell atlas assay, we observed a marked increase in peripheral myeloid‐derived cells post‐WMH. To elucidate the alterations and roles of these myeloid cells in the context of WMH, we executed a sub‐cluster analysis. This partitioned the myeloid cells into six distinct clusters, which were subsequently identified via SingleR as monocytes (specifically cluster 3), macrophages (clusters 1, 2, and 5), and dendritic cells (DCs; clusters 4 and 6). A pronounced proliferation of macrophages was observed in the WMH group, whereas monocytes were primarily observed in the sham group (Figure 5A,B). Such findings imply a potential transformation of striatal region monocytes into macrophages following WMH. To substantiate our prior cell typology determinations, we evaluated the expression of hallmark genes associated with specific sub‐clusters, such as *CD14* for monocytes, *Mrc1* for macrophages, and *Flt3* for DCs (Figure 5C). Moreover, we cataloged the top 10 marker genes for each cell sub‐cluster (Figure 5D). In our quest to discern the functional disparities among myeloid cells, we initiated a comparative functional analysis between the WMH and sham groups. GO enrichment analysis of the upregulated genes within the myeloid populations highlighted that the WMH group's myeloid cells exhibited heightened chemokine receptor expression coupled with amplified chemokine activity (Figure 5E). Moreover, a GSVA of pathway scores across each sub‐cluster revealed that pathways crucial to ferroptosis events, including oxidative phosphorylation and diverse fatty acid metabolic processes, were significantly activated in macrophages belonging to clusters 1 and 2. These macrophages were predominantly observed in the WMH group (Figure 5F).

### 3.6 Macrophage cell heterogeneity analysis

**FIGURE 6:**
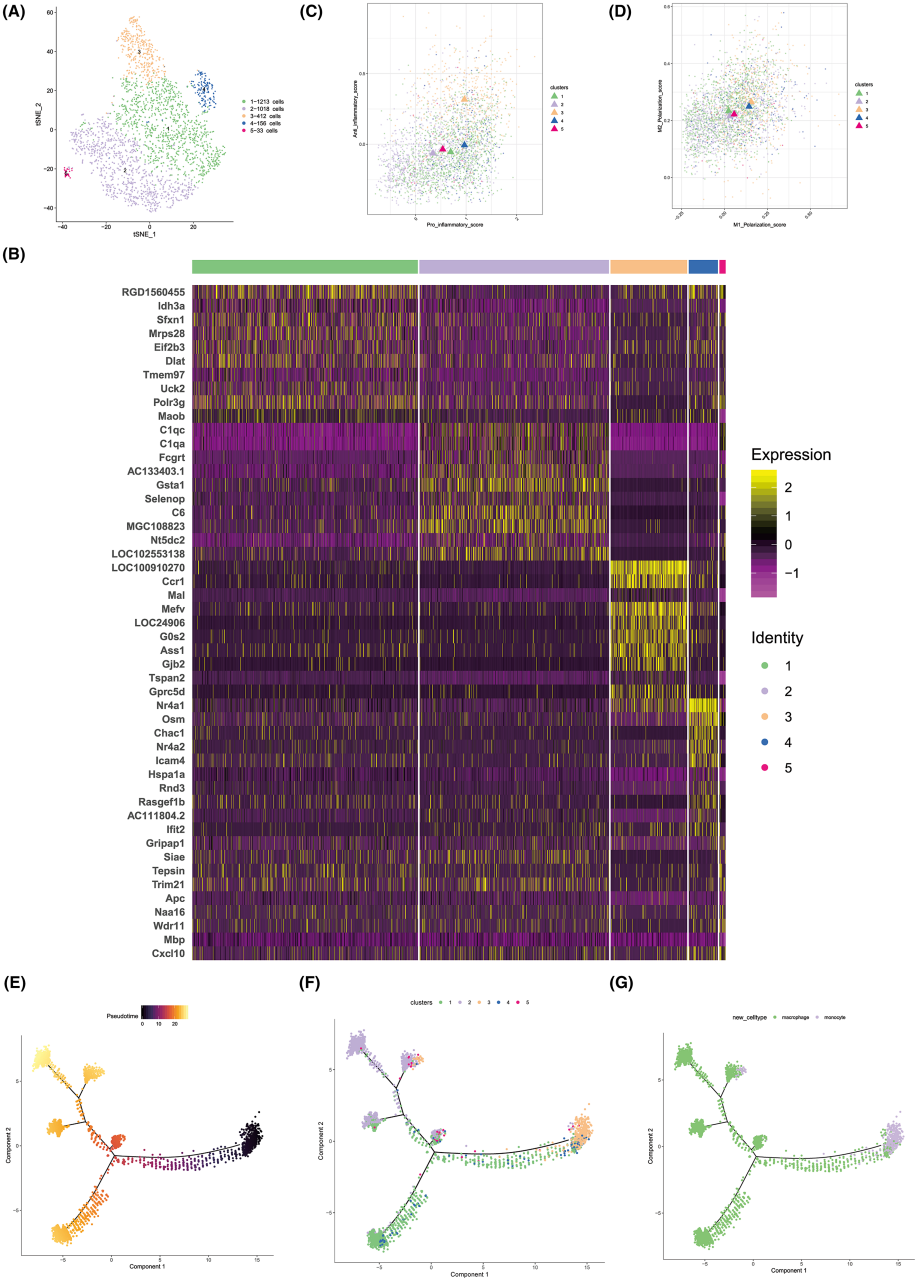
Comprehensive heterogeneity analysis of macrophage cell subtypes. (A) Sub‐classificatory assessment segregating monocytes and macrophages; Cluster 3 is identified as monocyte while the remaining clusters are categorized as macrophages. (B) Identification and enumeration of the top 10 marker genes distinctive to each of the five macrophage subclusters. (C, D) Functional analysis delineating the polarization states of M1 and M2 macrophages, encompassing both pro‐inflammatory and anti‐inflammatory characteristics. (E, F) Branched pseudotime trajectory analyses with each cell color‐coded by its pseudotime value (E) and by its designated Seurat clusters (F). (G) Single‐cell trajectory analysis elucidating the evolution of monocytes towards macrophage differentiation, highlighting Cluster 1 and Cluster 2 as principal contributors to pro‐inflammatory responses in the WMH cohort.

Given that the proliferation of macrophages is intrinsically linked to inflammation, we delved deeper into the sub‐clusters of macrophages and monocytes. This categorization subdivided the cells into five distinct clusters (Figure 6A). An analysis of the top 10 marker genes for each cluster (Figure 6B) revealed that cluster 3 represented monocytes, while the remaining clusters characterized various macrophage populations. To glean deeper insights into the proportions of M1 (pro‐inflammatory) and M2 (anti‐inflammatory) macrophages within these clusters, we employed gene set scoring. Our analysis suggested a predominant anti‐inflammatory macrophage phenotype across the clusters, with the notable exception of the monocyte‐rich cluster 3. Specifically, cluster 4 exhibited a pronounced anti‐inflammatory profile (Figure 6C,D). To map the developmental trajectory of macrophages, we implemented a single‐cell trajectory analysis focusing on monocytes and macrophages. This revealed a bifurcated developmental pathway from monocytes (cluster 3) transitioning into macrophages: one leading to cluster 2 macrophages and the other branching to clusters 1 and 4 macrophages. Intriguingly, cluster 1 seems to represent a transitional state of cells evolving from monocytes to macrophages post‐WMH (Figure 6E–G).

### 3.7 Analysis of oligodendrocyte heterogeneity

**FIGURE 7:**
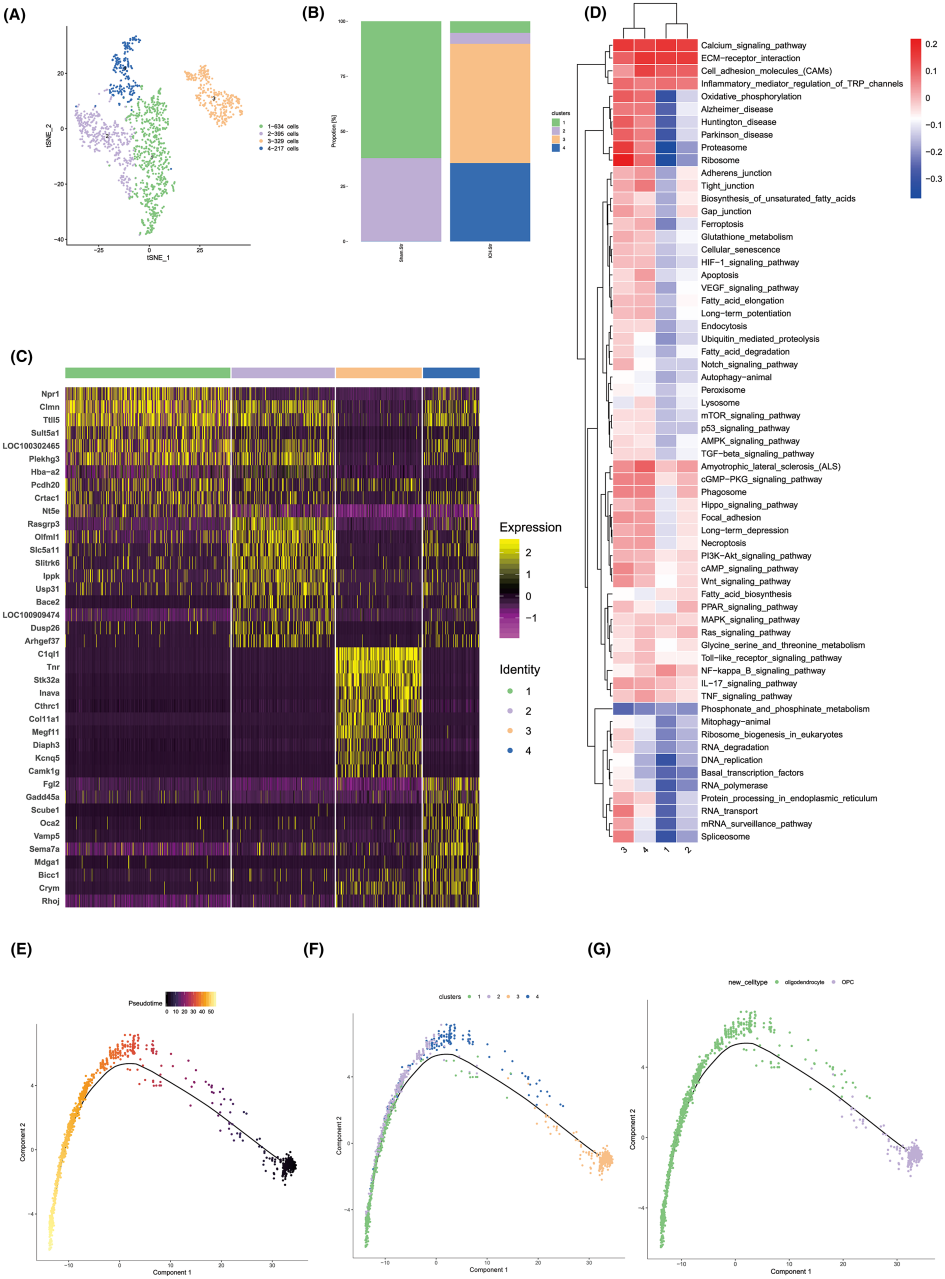
In‐depth heterogeneity analysis of OLGs. (A) Comprehensive subcluster analysis delineating the diverse OLG populations, accompanied by a proportional distribution across samples. (B) Histogram representation highlighting the proportional variance among OLG subclusters. (C) Enumeration and identification of the top 10 marker genes specific to each of the four identified oligodendrocyte subclusters. (D) Differential pathway enrichment as determined by GSVA across OLG subpopulations. (E, F) Branched pseudotime trajectory analysis, with each cell color‐coded based on its pseudotime value (E) and its associated Seurat clusters (F). (G) Single‐cell trajectory analysis elucidating the developmental progression from OPCs to mature OLGs.

OLGs are pivotal in sustaining myelin integrity, while OPCs serve as the forerunners to mature OLGs. Earlier observations identified a specific presence of OPCs within the WMH group (Figure 3F). Given this distinction, we embarked on a sub‐clusters analysis of both OPCs and OLGs. As depicted in Figure 7, cells in this category were predominantly segmented into four clusters, with cluster 3 representing OPCs. A proportional analysis revealed a predominance of clusters 1 and 2 in the sham group, whereas clusters 3 and 4 were more abundant in the WMH group (Figure 7A,B). Delving into the marker genes, we discerned an overlap of standard OLGs marker genes in clusters 1, 2, and 4. In contrast, cluster 3 prominently exhibited hallmark marker genes specific to OPCs, notably *C1ql1* and *Tnr* (Figure 7C). To delineate functional disparities, we undertook a GSVA for each sub‐cluster (Figure 7D). A comparative analysis between clusters 1 and 2 versus clusters 3 and 4 revealed distinct functional disparities. Specifically, clusters 3 and 4 demonstrated activation across a diverse array of signaling pathways, including but not limited to, ribosomal signaling, oxidative phosphorylation, cellular adhesion mechanisms, the formation of tight junctions, and endocytic processes. This suggests that post‐WMH, OPCs undergo a metabolic surge, priming them for the genesis of new myelin sheaths. Last, to map the developmental trajectory of these cell types, we employed single‐cell trajectory analyses, delineating the progression from OPCs to OLGs (Figure 7E–G). This suggests a compelling narrative: the WMH‐induced compromise of OLGs triggers an uptick in OPC numbers, which subsequently mature into OLGs. This proliferation and differentiation of OPCs post‐WMH potentially foster myelin regeneration in demyelinated brain regions, thereby facilitating WMH zone recovery.

## 4 DISCUSSION

The principal objective of this research was to construct a comprehensive single‐cell transcriptomic profile, aiming to shed light on the intricacies of cellular heterogeneity and interactions at the WMH site, based on a standardized animal ICH model targeting WMI. Historically, a plethora of methodologies have been employed in animal experiments to emulate human WMH. Sinar and Nahls,^27^ for instance, deployed a micro‐balloon expansion method to mimic post‐ICH hematoma; however, this model failed to replicate the effects of factors released by blood post‐ICH. Rosenberg adopted a strategy that introduced collagenase to induce ICH,^28^ where collagenase catalyzed the degradation of basement membrane and stromal collagen proteins. Yet, a consistent standard concerning the methodology, dosage, and timing of collagenase injection remains elusive in contemporary literature.^29^

While both collagenase VII and IV have been enlisted in formulating animal ICH models, the former predominates in usage,^30^, ^31^ with the latter being less frequent. Chen et al.^32^, ^33^ utilized varying doses of type IV collagenase to induce ICH in alignment with distinct experimental imperatives. For our study, it was imperative that our model rats exhibited neurological symptoms without excessive hemorrhagic lesions. The challenge was to identify an optimal model that authentically represents the hemorrhage dynamics of patients with white matter hemorrhage while ensuring repeatability. Our findings indicated that a dosage of 0.25 mU/kg type IV collagenase established a robust inflammation‐mediated white matter hemorrhage model that fulfills our experimental criteria. This model, which was consistent and reproducible, required approximately 20 min to completion.

Further, our investigation revealed post‐WMH sensorimotor function alterations in rats, contingent on the collagenase dosage. The influx of a significant blood volume into the brain tissue resulted in dose‐dependent hematomas, engendering mass lesions. Concurrently, a plethora of bioactive substances, including cell components, thrombin, and chemokines, were released, which in turn activated microglial cells (identified by Iba‐1). This process led to neuronal disintegration (marked by NeuN) in regions like the cortex and hippocampus, culminating in necrosis and an increased presence of inflammatory cells. Notably, MBP, a protein exclusively expressed in mature myelin sheaths, was found to be diminished in demyelinating lesions. On the contrary, SMI‐32, a non‐phosphorylated filament‐specific protein, becomes manifest post‐myelin damage.^34^ Our study affirms that white matter's myelin sheath underwent injury, marked by diminished MBP and elevated SMI‐32 expressions. Consequently, we posit that our collagenase‐induced inflammation‐mediated white matter hemorrhage model, particularly using 0.25 mU/kg collagenase, aptly mirrors the pathophysiological transformations post‐ICH, marking it as a reliable and precise quantitative model. The limitations of our histological techniques include variability in staining intensity and potential artifacts. We addressed these challenges through standardized protocols and multiple assessments to ensure accuracy. These methods, while robust, have inherent limitations in quantification.

In a subsequent study, utilizing scRNA‐seq, we delineated 17 distinct cell types, encompassing astrocytes, endothelial cells, microglia, myeloid cells, neurons, oligodendrocytes, OPCs, T‐NK cells, among others. Notably, a stark elevation in the proportion of myeloid cells was discerned within the WMH group. Conversely, the proportion of microglia, oligodendrocytes, and neurons appeared diminished. These alterations are indicative of the complex interplay between inflammation, cell death, and tissue repair mechanisms. The significant augmentation in the percentage of myeloid cells, coupled with the decrease in resident microglia and neurons, underscores the extent of inflammation and neuronal damage. This shift suggests a pronounced infiltration of peripheral myeloid cells, which could be contributing to the disease progression and severity. The increase in peripheral myeloid‐derived cells post‐WMH opens up new avenues for therapeutic strategies. Targeting these cells to modulate their activity could be a novel approach to mitigate the progression of WMH. This could involve strategies to reduce inflammation or promote reparative functions.

Within the microglial population, the sham group predominantly exhibited homeostatic microglia. However, post‐WMH, there was a transition towards microglial subtypes bearing unique transcriptional signatures, including those with pro‐, anti‐inflammatory, and proliferative tendencies, as corroborated by prior research.^22^ Despite an overall increase in the number of microglial cells, their proportional representation within the brain tissue actually decreases. Following a hemorrhagic event, a significant number of monocytes from the blood penetrate the brain tissue through the blood–brain barrier.^35^ This is due to the fact that the incoming monocytes constitute a substantial addition to the cellular landscape of the brain, thereby altering the overall cell population dynamics. Post‐hemorrhage, we observed a differential modulation in the populations of microglial subtypes, specifically M1 and M2 microglia. The M1 microglia, known for their pro‐inflammatory roles, show a continuous increase in number.^36^ Conversely, the M2 microglia, which are associated with anti‐inflammatory responses, initially increase but subsequently decrease in number.^36^ According to our previous study, we found that there was a same phenomenon in inflammatory polarization of microglia of white matter after Traumatic brain injury (TBI) that the expression of anti‐inflammatory M2 microglial cell genes is higher on day 3.^37^ Notably, around the 3rd day following a hemorrhage, there is a peak in M2 expression, indicating a pivotal moment when the expression of inflammatory pathways reaches its nadir. This reflects the transient predominance of anti‐inflammatory processes within the brain's microenvironment. This phenomenon can explain why inflammatory mediators such as IL‐17, TNF‐α, NF–κB, and Toll‐like receptors were found to be subdued during this period. Seven days post‐WMH, a downregulation of microglial M2 phenotype is observed, exacerbating the inflammatory response. This phenomenon underscores the necessity for regulation, rather than indiscriminate suppression, of microglial activity.

Of particular interest was the discernible surge in macrophages (a myeloid cell subset) within the WMH group, contrasted by a predominance of monocytes in the sham cohort. Prior investigations have emphasized ICH‐induced alterations in PBMC gene expression, particularly accentuating monocyte activation.^38^ Moreover, monocyte activation has been associated with metabolic processes including oxidative phosphorylation, fatty acid elongation, unsaturated fatty acid synthesis, and fatty acid degradation, predominantly identified within the WMH group. The augmented macrophage presence may facilitate the processing of erythrocytes and hemoglobin at hemorrhagic sites and engage in the catabolism of iron. This mechanism might inadvertently amplify oxidative stress, precipitating iron‐dependent cellular apoptosis. Hence, targeting macrophage activity, particularly their role in erythrocyte processing and iron catabolism, could mitigate oxidative stress and reduce iron‐dependent cellular apoptosis.^39^ Similarly, modulating monocyte activation, linked to various metabolic processes, might offer therapeutic benefits in managing WMH.^40^ This modulation needs to be finely tuned to balance the beneficial and potentially harmful effects of these cells. Targeting myeloid cells, particularly macrophages, and monocytes, could directly influence the inflammatory and metabolic pathways involved in WMH and ICH. This approach could lead to reduced inflammation, better management of oxidative stress, and improved outcomes in patients with cerebral hemorrhages. The effectiveness of targeting these cells would depend on the timing and specificity of the intervention. Early intervention post‐WMH, when these cells are actively involved in the inflammatory response, might be more beneficial. Early intervention might prevent the cascade of events leading to cell damage, but the specificity of targeting is crucial to avoid unwanted suppression of beneficial immune responses.

An examination of clusters 3 and 4 within the oligodendrocytes revealed activation pathways related to ribosomal signaling, oxidative phosphorylation, cellular adhesion and tight junctions, and endocytosis. This suggests that post‐WMH, OPC cells enter a heightened metabolic state, potentially priming for new myelin sheath formation.^41^ Single‐cell trajectory analyses hinted at evolution from OPC to mature oligodendrocytes, postulating that WMH‐induced damage to oligodendrocytes might trigger OPC proliferation and their eventual differentiation. This process represents a potential therapeutic target. Interventions that can enhance or support this natural progression could be pivotal in treating demyelination. Therapies could be designed to stimulate OPC proliferation or to facilitate their differentiation into mature oligodendrocytes.^41^ It sheds light on the reversibility of demyelination in WMH. By understanding the conditions under which OPCs differentiate into mature oligodendrocytes, it might be possible to reverse or mitigate the effects of demyelination. This could lead to significant advancements in the treatment of WMH and related conditions. Such a mechanism could be pivotal for myelin regeneration in demyelinated regions, promoting brain recovery. This aligns with Chu's findings,^42^ wherein both OPC proliferation and migration were enhanced in the adult ischemic brain, reflecting inherent remyelination and white matter repair endeavors. Cellular fate mapping, however, indicates that while OPCs in the ischemic brain do commit to the oligodendrocyte lineage, a significant proportion fail to mature into functional oligodendrocytes,^43^, ^44^, ^45^ thus potentially curtailing ICH recuperation over time.^42^ Importantly, this study augments our comprehension of cellular heterogeneity following ICH‐induced WMI in rats. Nonetheless, our analysis was confined to day 3 post‐WMH, emphasizing the need for extended temporal studies to holistically understand cellular dynamics and functional alterations. Future research endeavors in this direction are paramount.

Last, the alterations seen in the WMH group point to notable modifications in the cellular environment of the brain. These shifts are indicative of the brain's response to hemorrhagic injury and are likely to play a critical role in the progression and severity of WMH. Such as activated microglia and infiltrating monocytes. This suggests an enhanced inflammatory response in WMH, which is a key factor in the secondary injury processes following hemorrhagic events. Alterations in the proportions of neuronal and glial cells, including astrocytes and oligodendrocytes, point to the disruption of normal brain homeostasis and signaling. These changes could contribute to the loss of neuronal function and integrity, exacerbating the damage caused by WMH. The cellular changes observed provide insights into the mechanisms driving disease progression in WMH. For instance, the balance between pro‐inflammatory and anti‐inflammatory cells could influence the extent of tissue damage and repair processes. Understanding these cellular dynamics offers potential targets for therapeutic intervention. Modulating the inflammatory response or supporting the resilience and recovery of neuronal and glial cells could be key strategies in mitigating the effects of WMH.

## 5 CONCLUSIONS

In conclusion, we successfully developed a standardized WMH model targeting the white matter in rats. This model was authenticated through behavioral assessments, quantification of hematoma volume, and histopathological evaluations conducted over a span of 7 days. Employing scRNA‐seq, we delved into the cellular heterogeneity encompassing microglia, myeloid cells, macrophages, and oligodendrocytes. This investigation lays the foundational groundwork for elucidating specific cell types and molecular pathways implicated in WMH, paving the way for subsequent clinical and experimental inquiries.

## AUTHOR CONTRIBUTIONS

Guohua Wang, Qianqian Luo, and Hua Feng conceived, designed, and supervised the entire study. Lisha Ye, Xiaoyan Tang, Jun Zhong, Wenfeng Li, and Ting Xu performed the experiments, acquired data, analyzed data, and drafted the manuscript. Chao Xiang and Jianjun Gu participated in part of the experiments, acquired data, and analyzed data. Guohua Wang and Lisha Ye reviewed and edited the manuscript. All authors contributed to reading and approved the submitted version.

## FUNDING INFORMATION

This work was supported by the National Natural Science Foundation of China (Grants 82171190 and 81873924), Key Scientific Research Projects of Colleges and Universities in Henan Province in 2021 (Grant 22B310001), China Postdoctoral Science Foundation (2020M673649) and Nantong Municipal Science and Technology Project (MS12021020 and MS22021010).

## CONFLICT OF INTEREST STATEMENT

The authors have no conflicts of interests.

## Supporting information


Figure S1

Appendix S1

## Data Availability

The scRNA‐seq data underpinning the discoveries of this study are archived in the sequence read archive (SRA) database under the accession code PRJNA1005089. Other data that support the findings of this study are available from the corresponding author upon reasonable request.
