# Supplementary material for: Unraveling the complex pathophysiology of white matter hemorrhage in intracerebral stroke: A single‐cell RNA sequencing approach
Source: CNS Neurosci Ther. 2024 Mar 3;30(3):e14652. doi: 10.1111/cns.14652 (PMC10909628; doi:10.1111/cns.14652)
Supplement: Supplementary file 1 — Figure S1 [file CNS-30-e14652-s001.docx]

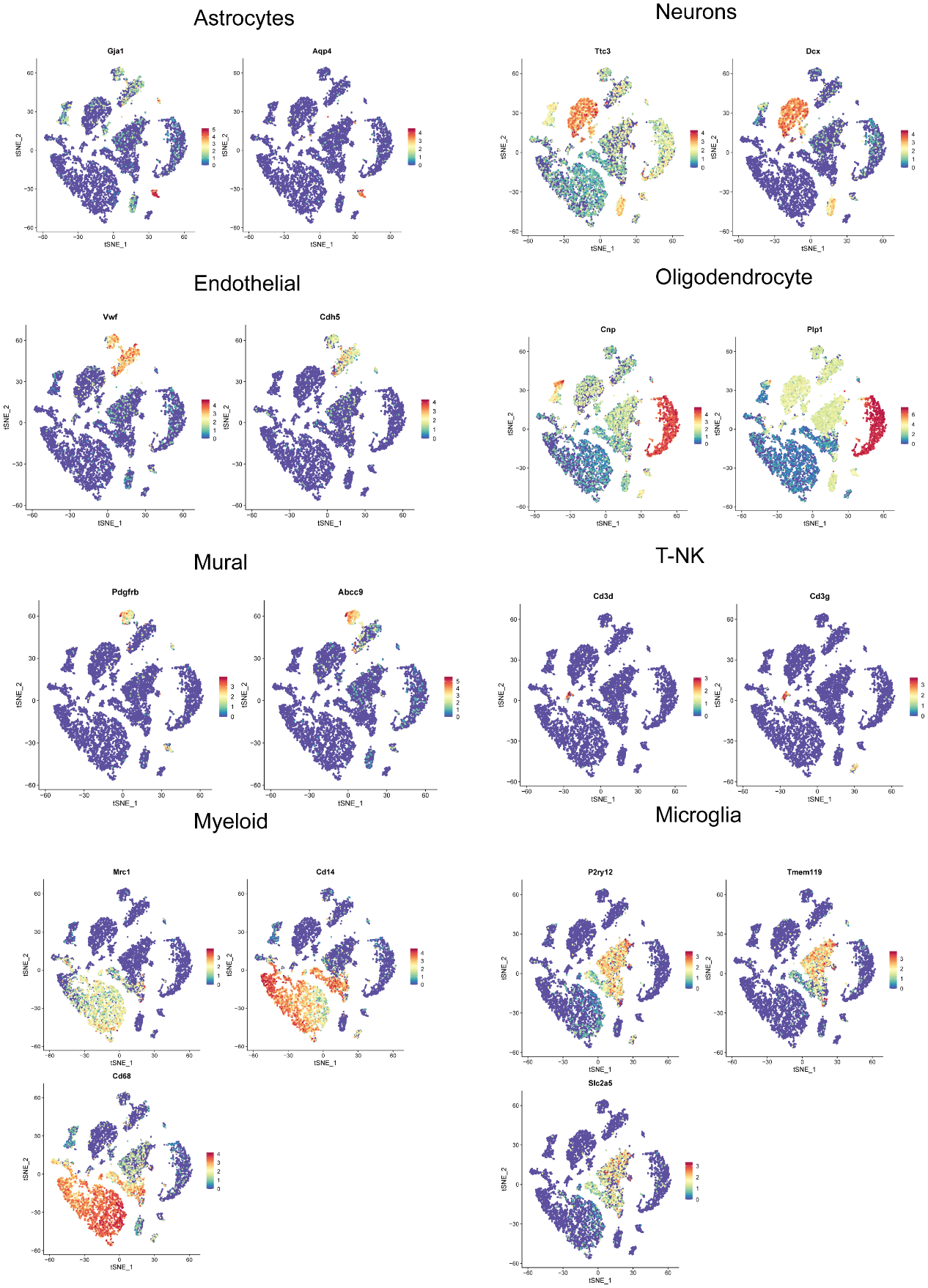


**Figure S1** Marker gene representation across diverse cell subsets. After rigorous quality control measures that filtered out subpar and duplicated cells, a total of 4,440 cells from the sham group and 4,806 cells from the WMH group were curated for in-depth analysis.





**Figure S2** Variations in microglial phenotypes following WMH. The expression levels of CD16, inducible Nitric Oxide Synthase (iNOS), CD206, and Arginase 1 (Arg1) in rat brain tissue were evaluated using quantitative Real-Time Polymerase Chain Reaction (qRT-PCR). This assessment was conducted on the 3rd and 7th days post-WMH. *n* = 6 in each experimental group; **P*<0.05, ***P*<0.01, ****P*<0.001 vs sham group.
