# Supplementary material for: Unraveling the complex pathophysiology of white matter hemorrhage in intracerebral stroke: A single‐cell RNA sequencing approach
Source: CNS Neurosci Ther. 2024 Mar 3;30(3):e14652. doi: 10.1111/cns.14652 (PMC10909628; doi:10.1111/cns.14652)
Supplement: Supplementary file 2 — Appendix S1 [file CNS-30-e14652-s002.docx]

**Quantitative real-time PCR (qRT-PCR)**

To assess gene expression in the striatal tissues of both sham and white matter hemorrhage (WMH) models, we employed qRT-PCR at 3 and 7 days following WMH. Extraction of total RNA was performed from the tissues, followed by cDNA synthesis. For the amplification of specific genes, we utilized primers for β-actin (a commonly used housekeeping gene), CD16, CD206, Arg-1, and iNOS. The primer sequences were as follows: β-actin Forward: 5'-GAGGGAAATCGTGCGTGAC-3', Reverse: 5'-GCATCGGAACCGCTCATT-3'; CD16 Forward: 5'-TTTGGACACCCAGATGTTTCAG-3', Reverse: 5'-GTCTTCCTTGAGCACCTGGATC-3'; CD206 Forward: 5'-CAAGGAAGGTTGGCATTTGT-3', Reverse: 5'-CCTTTCAGTCCTTTGCAAGC-3'; Arg-1 Forward: 5'-TCACCTGAGCTTTGATGTCG-3', Reverse: 5'-CTGAAAGGAGCCCTGTCTTG-3'; iNOS Forward: 5'-CAAGCACCTTGGAAGAGGAG-3', Reverse: 5'-AAGGCCAAACACAGCATACC-3'. Under standardized conditions, the qRT-PCR was executed, and the expression levels of the target genes were normalized to β-actin. Data analysis was performed using the 2^-ΔΔCt^ method to compare gene expression between the groups and time points, providing insights into the molecular mechanisms underlying white matter injury and recovery.
